# “The Razor’s Edge of Timing:” A Phenomenological Analysis of Decision-Making Processes Surrounding Medical Aid in Dying

**DOI:** 10.3389/ijph.2024.1607435

**Published:** 2024-08-30

**Authors:** Jennifer Currin-McCulloch, Nathan Gallo, Yixuan Wang, Kim Mooney

**Affiliations:** ^1^ School of Social Work, Colorado State University, Fort Collins, CO, United States; ^2^ Practically Dying, Inc., Longmont, CO, United States

**Keywords:** values, medical aid in dying, phenomenology, end-of-life decision making, timing

## Abstract

**Objectives:**

The study aimed to explore how terminally ill individuals in the United States approach medical aid in dying (MAID), including personal, interpersonal and structural factors that influence their decision-making processes.

**Methods:**

This embodied phenomenological study incorporated semi-structured (N = 9) interviews with seven terminally ill adults who received a prescription for MAID. Interviews occurred over Zoom between October 2021-January 2023 and was guided by Ashworth’s framework for exploring phenomenological lifeworlds. Participants were invited to share perceptions of their lifeworlds in pursuit of MAID including values; embodied health, ability, and emotions; space and place in society; reflections on time/timing; and political and cultural discourse. Data analysis integrated Wertz’s phenomenological psychological analysis methods.

**Results:**

The phenomenon of choosing MAID is an intricate juggling of lifeworlds between participants’ embodied relationships, values, time and agency which lead to co-existing experiences of uncertainty and hard-won relief.

**Conclusion:**

Our findings contribute cutting-edge knowledge of the decisional tensions and triumphs terminally ill individuals encounter as they approach MAID and highlight practical implications for health and mental health providers in preparing psychoeducational support for those seeking MAID.

## Introduction

Two centuries of progress in modern medicine and public health education have led chronic and degenerative disease to outpace infectious disease, paradoxically leading to an increase in slow, protracted deaths of old age, organ failure, or frailty [1–3]. End-of-life (EOL) healthcare options may foster extended life, yet increase the likelihood of physical distress [4, 5] and psychosocial strain [6–10], such as decisional anxiety [11]. How one chooses to spend their final days and approach EOL decisions depends on access to resources (e.g., educational, financial, social, physical and cognitive ability) [12–14] and personal values surrounding health and quality of life [15, 16]. Research documents the complex decisional calculus that terminally ill individuals balance between layers of personal, interpersonal and structural factors [17–19]; however, evidence describing the phenomenon of choosing medical aid in dying (MAID) in the United States remains insufficient.

The 21st century’s primary social challenge of dying — the timing of death — focuses on prevention of traumatic deaths, dependency, and uncertainty [20] in favor of more humanitarian dying processes [21]. In the late 1980s, residents of western U.S. states advocated to legalize MAID as an EOL option that promoted choice and agency [22]. Oregon residents successfully led a voter ballot initiative to legalize MAID in 1997 [23] and 9 other U.S. states and the District of Columbia have followed suit [24–26]. Requirements to access aid in dying vary; however, each U.S. state and the District of Columbia require that terminally ill individuals have cognitive capacity and receive a prescription from a medical provider for medications they will self-ingest to end their lives [24, 27, 28]. Kozlov and colleagues [29] compiled data from districts’ disparate registries to determine that over the past 23 years, 8451 terminally ill individuals received MAID prescriptions, while 5,329 died using these medications. Among these individuals, the median age was 74 years, and the majority were male, non-Hispanic White, college educated and had a diagnosis of cancer. While budding data demonstrates trends in usage, limited research provides context around forces that drive terminally ill individuals’ decision-making to pursue medical aid in dying in the United States [30, 31].

Decisions at the EOL often involve painful trade-offs, regardless of an individual’s degree of preparation [32, 33]. Values—and the strength of their clarity—act as psychological guideposts for terminally ill individuals to choose a path forward amidst uncertainty [34, 35]. Culture frequently serves as a constraining factor, narrowing the possible values that influence a decision [36–38]. Overwhelmingly, individuals who have chosen MAID report basing their decisions on the values of autonomy, control, and choice [39, 40]. However, we lack broad understanding of how and why people in the United States integrate these values into their aid in dying decision-making processes [41, 42].

Knowledge from other countries about decisions to choose MAID echo terminally ill individuals’ desire to limit anxiety surrounding potential suffering and the loss of personal dignity [43, 44]. Notably, uncertainties surrounding MAID access and numerous medical tasks [43, 45] make it impossible to avoid decisional anxiety. Given the estimated 74 million people living in United States’ jurisdictions with aid in dying legislation [24, 29], an urgent public health imperative remains to understand decision-making processes, benefits, and barriers residents encounter.

### Study Purpose

This study aimed to explore how terminally ill individuals in the United States approach a medically assisted death, including (1) personal, interpersonal and structural factors that influence their decision-making processes; (2) values about their body and identity; and (3) pivotal physical, psychosocial and existential moments throughout the preparation for an aid in dying death.

## Methods

### Research Design

Maurice Merleau-Ponty’s phenomenology of perception served as the philosophical framework to inform the study design, data collection, analysis, and presentation of findings. His embodied phenomenology incorporates a lens from which to classify one’s perceived experiences of their body within a larger social context [46] and provides a framework from which to conceptualize the interdependent, layered contexts of the body, interpersonal relationships, and social structures inherent in an aid in dying death [47].

### Sample

The study’s sample (see Table 1) included seven adults, ages 58–94 (mean age 73.1) who met MAID criteria and had received a prescription for aid in dying medications. Purposive sampling entailed partnering with medical and mental health providers and organizations that provide resources for those gathering information about MAID (e.g., the American Clinicians Academy on Medical Aid in Dying, Compassion and Choices, Death with Dignity, doula organizations, the Association for Death Education and Counseling, and state-based aid in dying support and advocacy organizations). We took a broad approach to recruitment by partnering with providers and support organizations from different states, and personal interests in MAID so that we could reduce bias in our recruitment process. Snowball sampling happened when two participants shared about their research participation with friends. Recruitment sources shared a flier (in English or Spanish) with interested individuals that included a link and QR code to the online consent form.

**TABLE 1:** Participant sociodemographics (N = 7) (United States, 2024).

Participant number and pseudonym	Age	Terminal illness
P4 Cheryl	58	Gynecological cancer
P17 Dorothy	89	Heart failure
P20 Linda	61	Motor neuron disease
P30 Nancy	71	Breast cancer
P35 Mary	59	Gynecological cancer
P45 George	84	Parkinson’s Disease
P54 Lorraine	94	Heart failure

To protect the confidentiality of participants, of whom several chose not to tell loved ones about their pursuit of MAID, we present participant sociodemographic data in broad terms and identify quotes with a pseudonym. The sample was predominantly female (6/7, 85.7%), White (100%), and worked in health, mental health or education fields. Their terminal diagnoses included cancer (3/7, 42.8%), neurological illness (2/7, 28.6%), or cardiac disease (2/7, 28.6%). Participants represented five of the 11 authorized jurisdictions, all of which have disparate access to MAID.

### Data Collection

Prior to data collection, members of the research team participated in individual and group-based reflection to begin the process of bridling [48] to decipher how their prior knowledge, practice and potential biases as a healthcare social worker, researcher or thanatologist may inform data collection, analysis and the presentation of the findings. After completing the Qualtrics online consent form, survey questions addressed participant’s health status and when they began the MAID process. Each participant consented to have their interview both audio and video recorded over Zoom between the months of October 2021 - January 2023. Two participants chose to complete one follow-up interview within 3 weeks of their initial interview. Caregivers who joined the interview also completed consent documents. Participants received a $30 Amazon egift card for each interview.

Initial interviews followed the same semi-structured interview guide (see Table 2), with the follow-up interview guide gathering updates on their experiences in approaching an aid in dying death. The initial interview questions focused on personal, interpersonal and structural influences on their decision-making, and barriers and triumphs encountered throughout their pursuit of MAID. During the two follow-up interviews, the participants delved more deeply into their decisions about the timing of their final day, preparation of legal documents, tensions they felt in prioritizing their needs over their loved ones’ needs, and personal values that appeared salient in their decision-making. Additionally, the second interview helped us to better understand how decisions emerge within the complexity of illness uncertainty, and how the progression of disease, inner self-dialogue, interpersonal communication, and other factors change over time. Interviews occurred on Zoom and lasted between 35–93 min (average 52 min). An initial transcript was produced by Zoom and edited by the second author while watching the video to enhance accuracy.

**TABLE 2:** Semi-structured interview guide (United States, 2024).

Interview topic	Prompts
Introduction to the interview process	*We’d like to begin by thanking you for taking time to share your story with us. It is an honor to learn from you about your experiences surrounding your decision to utilize MAID. There may be times in our conversation that may bring up difficult thoughts. I would like for you to let us know if you need to stop and take a break, grab some water, or reschedule for a later time*.
If okay with you, we would like to start by learning a little bit about you and your medical condition.	Knowing that individuals are not defined by their diagnosis, can you please share with us about yourself? Who are you? Family? Work? Hobbies? Can you tell me about your illness and when you learned that it was not curative?
Please walk me through what the decision-making process was like for you when you were considering medical aid in dying?	How did you learn about MAID? What was your daily life like before you made the decision to choose medical aid in dying? Where did you receive your education regarding MAID? Discussion? Literature? Video? Internet? If through a healthcare provider(s), which one(s)? What/who was the most supportive at the time of deciding whether to choose MAID? After making the decision? Did you experience any barriers or changes in relationships (professional or social) after making this decision?
We would love to learn a little bit about what life has been like for you since you chose medical aid in dying.	How would you describe the practical and/or emotional support you have received (or haven’t received)? Who, if anyone, has been your most reliable support since you started your journey with MAID? Is there a type of resource or support you needed that you have yet to receive? What/who was/were the greatest barrier you encountered since deciding to utilize MAID?
What are your hopes for the last days of your life? How do you picture it?	Have you chosen the day you will take your aid in dying medications? Please share with us what you envision your final day to be like Who will be there? Where will you be?
Before we end, we would like to ask if there is anything that we haven’t addressed that you feel would be important in understanding your experiences or that of your loved one in relation to medical aid in dying?	
	
Interview closure and expression of gratitude	*Thank you for sharing your story with us today.*

### Data Analysis

The first three authors participated in data analysis, incorporating Finlay’s [49] approach to entering into a phenomenological mindset and Wertz’s [50, 51]and Ashworth’s [52] methods for analyzing phenomenological data. Prior to coding data, the first three authors individually read all nine transcripts to discern the overall landscape of the data. Each individually coded the first interview and then met as a team to discuss initial thematic insights. Ashworth’s [52] lifeworld framework guided our monitoring for how participants portrayed their (1) values; (2) embodied health, ability, and emotions; (3) space and place in society; (4) reflections about temporal factors; and (5) political and cultural discourse surrounding their pursuit of MAID.

Following Wertz’s [50, 51] guidance for phenomenological psychological analysis, we completed the following four analytic steps: (1) determined general experiences within individual interviews; (2) compared experiences across individuals to discern general and disparate experiences in approaching MAID; (3) performed imaginative variation to ascertain invariant meanings among participants’ experiences; and (4) developed an overall framework to represent the phenomenon of terminally ill individuals’ decision-making experiences in pursuing MAID in the United States. Within the context of this study, imaginative variation entailed our team freely imaging which aspects of the decision-making process about MAID are essential to the experience of MAID decision making and, if removed, which parts of the participants’ experience are not essential to the phenomenon. For instance, we discussed the differences in illness uncertainty around the timing of being able to self-ingest aid in dying medications among those with malignant and nonmalignant terminal conditions. We freely imagined which factors of the experience within and across participants with malignant and nonmalignant conditions were essential to the phenomenon of MAID decision-making and which factors, if removed, did not appear to change the overall phenomenon of illness uncertainty around self-ingestion.

### Trustworthiness

The study incorporated numerous quality checks through the study design, implementation, data analysis and presentation of findings [53, 54]. Our process for multicoder data analysis included individual readings of all interviews and meeting together weekly throughout the data analysis phase to discuss coding processes. When disparities in coding occurred, we discussed nuances until we reached consensus. We created a matrix using a qualitative software program, MAXQDA, version 2022 [55] to track development and revision of lifeworld experiences, code and meaning saturation [56], and meaning-based and analytic memos. The aim of phenomenological studies is to pursue saturation across cases instead of within cases [57] and to gather an understanding of commonalities, and different interpretations based on different lived experiences [58]. Based on the participants’ energy levels and willingness to share, we spent at least 30 min and up to 180 min with participants. This allowed us to gather an expansive understanding of their nuanced and common experiences in pursuing MAID. We ensured that all participants’ common understandings and individual explanations of MAID decision-making were adequately coded through ongoing independent coding and group discussions until no new codes/themes emerged.

## Results

The phenomenon of approaching medical aid in dying in the United States is a complex life process that involves an intricate intertwining of one’s relationship with their body, time, values, and relationships with others and systems, including legal or policy requirements based on their space/place in the world. Although separate lifeworlds in the experience of seeking and preparing for aid in dying, each appear interconnected and dependent on the body in its terminal state.

### Living in a Terminally Ill Body

The decision to pursue MAID evolved after months to years of health uncertainty living in physical frames which no longer supported their ability to pursue life’s purpose or essential activities of daily living. Habitual physical activities now required intentional planning and left participants yearning for bodily transparency and certainty that their bodies would sustain function in the coming days. Their bodies became “jackets” (Linda) or shells that housed vital organs with minimal functionality in the real world. For some, their physical decline was gradual and transparent, while others lived with constant uncertainty of when their bodies would reach a point where they “are in a coma parked in a corner of the room” (Nancy). A physician with keen knowledge of the body portrayed this embodied tension: “It’s a regular downhill course but it doesn’t tell me when it’s going to end” (George). Distressing physical symptoms weighed heavily in choosing MAID, including excruciating muscle and nerve pain, cancer’s spread to vital organs, loss of feeling or control of limbs, and declining ability to breathe or swallow.

Eventually each participant reached a relationship with their body where they deemed it expedient to pursue aid in dying. Those with cancer commonly pursued curative or stabilizing cancer treatments but made conscious decisions to stop cancer therapy when the suffering from treatment-related side effects exceeded the benefits of life extension. As portrayed by a woman dreading another excruciating malignant ascites abstraction, “I can’t do any more treatment and I just can’t. My body will not allow me. My body was screaming, ‘No. No more’” (Cheryl). Unmanageable pain emerged as paramount in her decision to end chemotherapy. A palliative care physician with a terminal cancer shared her newfound insights on the limitations of pain management:

[Hospice] can be a really good way to die. And then to find out myself that I had this pain that could not be managed really made me realize that there were probably many deaths that were much more uncomfortable than I ever appreciated (Mary).

Of note, the majority of individuals chose to discontinue curative medical treatments and enroll in hospice services.

### Dwelling in the Tension Between Self and Others

At the end of their lives, illness robbed participants of the physical function necessary for maintaining social roles. For example, Linda chose MAID primarily because the disease had forced her to give up a career that gave her purpose and transformed her interactions between “wife and husband” into “patient and caregiver,” leaving a sense of crisis in fulfilling her role as a family member. For most participants, the inability to perform social roles seriously threatened their identity and sense of worth as well as their perception of being “useful” (Lorraine) to others and to themselves.

Socioculturally and historically constructed memories associated with death influenced how participants defined and chose a “good death.” Nancy did not want to be “stuck like a rock in the corner” like her mother, and Mary yearned to avoid going through the “awful death” she saw some of her family members experience. Both participants chose MAID to gain control of what their last days would look like. Death-related memories also led many participants to consider what kind of legacy they would leave behind, like the image of a healthy mother (Cheryl) or being a change-maker through sharing their MAID story (Linda). Additionally, participants’ narratives included pleasant memories of the past, such as family trips (Lorraine), successful careers (Linda), and days of health and vitality (Cheryl), which reduced their regret at facing death but led to lower satisfaction with their current quality of life.

Choosing MAID meant relief for most participants, but for their families and friends, a range of meanings emerged, including the loss of a loved one, a betrayal of faith, and a decline in hope, creating a conflict over their shared meaning. For example, Dorothy declined to tell all of her family and friends about her choice because she worried they wouldn’t accept it. Frequently, loved ones gained a shared understanding of the decision to pursue MAID by witnessing participants’ suffering. Cheryl’s children unexpectedly supported her choice after observing the ravages of chemotherapy’s side effects.

Illness caused shame and embarrassment for most participants because they were not able to control or present their bodies as healthy people and, therefore, no longer looked “normal” to others; thus, exacerbating their sense that reality is uninhabitable: “You might leave a puddle of water at your table on the floor from your leaking leg, and nobody knows what the hell is wrong with [you]. So, [I] know about bathrooms. I mean, it’s not pleasant at all” (Dorothy).

Compared to dying naturally or “fighting” until the end, MAID is not universally embraced by society because it is not seen as a socially sanctioned way to approach one’s dying process. The resulting controversy and stigma undermined participants’ sense of the legitimacy of their choices and added pressure to their decisions and psychosocial wellbeing at the end of life.

### Perceiving Time in an Ill World

The perception of internal and external countdowns forced participants to the “razor’s edge of timing” (Linda), increasing their decisional urgency. Their internal decline led to decreased bodily function; the bad days gradually outnumbered the good ones. As Nancy explained, “I’m tracking my body, I can see that it’s going down, I can see, this would be the optimal time to take this drug.” For elderly participants, aging compounded the loss of functionality. Dorothy also shared, “You’re too old, you can’t … if you fixed any part of it, there’s still three more things that aren't working right, so what is the point in hanging around.”

Externally, participants received evidence from a third perspective, their terminal prognosis, that that death was indeed imminent. In contrast to participants’ embodied perception of time, medical professionals perceived time more objectively. Nancy explained, “When I talked to my nurse through hospice, she was saying, ‘Well, you probably have a month to live or maybe a month and a half.’”

The legal requirements for MAID created tension around timing death. Although they were able to have more control over their deaths, participants commonly worried about losing control if their cognitive or self-administration abilities diminished. Therefore, every person had to spend their last days constantly surveilling their physical condition. As Nancy said, it was like “a crazy dance.” Moreover, they had no control over how the disease interacted with other concurrent life events on the timeline, adding complexity to their decisions. The COVID-19 pandemic led Dorothy to worry that the virus would erase her ability to choose MAID.

The disease also deprived the participants of temporal possibilities, driving them to make proactive decisions rather than wait for the hand of fate. They could not go back to their past identities. As Lorraine shared, “After the stroke, I decided I’m not ever going to be able to do the things that I’ve done before.” The present moment was no longer bearable because their body could no longer support them. Dorothy described, “Living like this is not living, so I’m ready to go.” Parallel to this, the future was no longer accessible as their days became numbered. Although Cheryl wanted to stay and spend Christmas with her loved ones, she believed that cancer would not allow this family celebration.

### Values as Anchors in a Shifting Situation

Values were foundational to decision-making throughout the MAID process, enabling participants to make deliberate decisions amid profound uncertainty, especially around eligibility, receipt of medications, and timing. Each participant understood MAID as an alternative to a natural death—an option—instead of the only path forward and anchored their decisions in spoken values of personal agency and control, even in the face of conflicting priorities. Cheryl explained, “If you decide you don’t want to use it, you don’t have to, and that was really key for me. Because to be honest, doing this really goes against all of my spiritual beliefs.” Mary acknowledged MAID as “another way” that “advance[s] the process that’s happening anyway,” finding a profound sense of relief in preserving the capacity to choose in a position of limited options, or perceivably none at all.

Some wrestled with the consequences of shifting values while actively making choices in a dying body. Nancy described MAID as “scary,” saying that “it’s not who I am as a person generally. I still am coming to terms with that reality.” Asked what she meant, she responded, “I am not a person that would do suicide or kill myself.” Individually, the body’s biological processes marched on, and values that undergirded decisions came under pressure; most explained that either they could choose MAID or death would choose them. In light of this, Lorraine described the importance of control with conviction and reprieve: “I’ve made my decision and I’m not changing my mind.”

A perpetual value tension hovered over controlling the day of death, framed frequently as a trade-off. George shared, “you also need to understand what you are giving up by staying and what you are giving up by dying.” Control sat uneasily among other personal sources of meaning, as he explained:

By dying, you lose the pleasure of relating to people, special talks, books, wonderful kindness of your caregivers. You don’t want to leave each other. By living, you are also losing connections and important people to you. You have the symptoms which brought you here to start with.

Despite this, decisions were bearable as participants held irreconcilable values and existential unease that often persisted until their Last Day.

### Agency as a Tool to Navigate Social (Mis)Fortune

All seven participants acknowledged a combination of social advantages contributing to their agency and ability to come to the final decision. Popular rhetoric emphasizing a patient’s own choice and dignity was complicated by stories of MAID constrained by external factors, including legal, economic, social, temporal, and relational demands. Mary labeled MAID an “entitled service” while Cheryl called it a “luxury,” having had ample time to research the intricacies of the journey. Despite this, she shared, “So you know, I went through hell. The MAID process is not exactly easy. They make it really annoying,” hinting at the time, energy, and stamina required.

Connections to the healthcare system were vital to spurring the decision-making process forward. Plans were made with the help of “insider” information from clinicians across the medical hierarchy, typically through physicians connected to supporting organizations or with previous histories supporting MAID. For example, Mary’s primary care doctor, barred by law to discuss the option in a federal clinic, referred her to a palliative care clinician and comfortingly, “The process was incredibly streamlined from there on, once [they] knew who to talk to.” Linda, despite “anger and rage at the machine” in securing a formal diagnosis for her neurological illness, found available physicians through *Compassion & Choices’* website.

Accessing the legal option and final medication “cocktail” depended largely on securing a place in a MAID-eligible, safe location. Although most of those living in facilities were forced to find alternative locations to self-administer aid in dying medications due to facility policies, a few found a ready place in a family member’s home: “My daughter said we could do it at her house, but we had other choices.” (Lorraine). Others confronted more distressing alternatives. Linda spoke with frustration about how her hand was forced, relocating from the state where she and her children were raised. Holding up photos of herself perched on a bridge between two state lines, she reflected on the irrationality of legal availability:

So, I’m standing on the river because I taught geography for many years. There is this, there is this arbitrary body of water that has changed course over the millennia many times and because I’m on one side of it, I could not access what I would have liked (Linda).

For each person, the right state and physical spot became the difference between a plan and a hard-won, relieving reality.

## Discussion

Findings from this study provide novel understanding of the layered context of decision-making involved with the pursuit and ingestion of aid in dying medications in the United States. We reveal the decisional calculus that terminally ill individuals factor between aspects of their five lifeworlds‐‐lived body, social space, temporality, values, and agency‐‐as they plan for death. In addition to a congregation of physical symptoms, emotions ranging from relief and hope to anxiety appeared across all five lifeworlds.

MAID presents opportunities to circumvent the arrival of social death before physical death, which enables the intentional choreographing of death before individuals are unable to recognize themselves, in line with the Western sociocultural construction of a “good death” [59, 60]. In practice, however, medicolegal structures underlying MAID in the United States do not permit complete independence and control; instead, they require solid social relations to support their desires [59]. The results of this study illustrate that divergent interpretations of MAID can provoke meaning-sharing conflicts between participants and their loved ones. The perception of MAID as a social and ethical stain turns the practice into “dirty work” for some healthcare providers [61], (p535) reinforcing stigmas that arise when deviating from “normal” [62]. Thus, adding obstacles to terminal patients’ legitimate healthcare claims and unjust social pressures during their precious final days.

In the world of the terminally ill, their embodied experience of time overshadows objective time on a calendar [63, 64]. Choosing MAID adds temporal tension, that is, fearing that the timing of death would occur too soon or too late. Furthermore, our findings echo existing research: a happy past is distant, a focused present is narrow, and a limited future is unpredictable [63–65]. We identified that participants’ perceptions of time were informed by their lived experience of their body’s shrinking ability; thus, highlighting the imperative for healthcare providers to be attuned to and deliver care that is in synch with their patients’ needs [63].

Values and medical aid-in-dying scholarship have historically focused on perspectives of healthcare workers [66–68], family members [69–72], and the public [73, 74]. Our study complicates simple notions of aid-in-dying as a person’s choice and way to maintain one’s dignity [75]. Anchored values like control and choice competed with other valued aspects of their lives, including their loved ones, spirituality, and the meaning attached to living another day.

Participants’ stories extend existing research on the relationship between the EOL and “relational autonomy” [17, 76, 77] that account for factors outside of an individual’s control, especially during a time of heightened dependence. Findings strengthen layered accounts of participants needing to receive substantial help as they navigate obstacles posed by bureaucratic guidelines, inconsistencies in the medical system, and structural disadvantages [31, 78, 79].

### Implications for Practice and Policy

Participants’ narratives highlight opportunities for advocacy, support and care systems to reduce tensions of terminally ill individuals seeking MAID, including the development of psychoeducational resources to prepare them for potential stress resulting from bodily uncertainty and structural and social barriers they may encounter in preparing for self-ingestion. Ideally, those seeking MAID and their caregivers could have access to peer support groups before and after a MAID death to normalize their experiences, gather guidance, and find emotional support through this often disenfranchised process. Providers should proactively educate and prepare patients and loved ones about the potential for psychological distress around the timing of death. Healthcare teams can share checklists or videos for their clients to help them prepare for the tasks leading up to and including the day of death, such as local death doula resources, MAID support organizations, or former families that they have worked with who would be willing to mentor others beginning the MAID process. Many checklists address medical tasks and the additional of psychological and existential factors to be aware of may also be of benefit. Psychoeducation and support programs like Washington and Oregon’s End-of Life and other state non-profit support organizations can assuage logistical fears and normalize the strains individuals face preparing for a MAID death.

From a macro perspective, the revision of state and federal policies may be influential in reducing the distress experienced by those seeking MAID. Pope [80] details ten examples of macro approaches to reduce barriers to MAID access in the U.S. A few examples include proposing legislation to shorten or waive the mandatory waiting periods between terminally ill individuals’ requests for MAID and enabling advanced practice nurses to serve as authorizing providers.

### Limitations

Limitations from this phenomenological study include the narratives reflecting solely terminally ill individuals’ perspectives. Additionally, the phenomenon represented here portrays the perspectives of those living in the U.S.; those living in other countries with more expansive eligibility requirements may portray their decision-making about an aid in dying death differently. Two participants’ follow-up interviews afforded us the opportunity to discuss in-the-moment decisional changes, while the other participants retrospectively recollected changes. Participants brought heterogeneous physical experiences, yet had similar race and ethnicity, educational attainment, and access to financial and health resources. Future MAID research should integrate diverse participant narratives to understand decision-making considerations among those from socially or financially marginalized groups, as well as terminally ill individuals’ loved ones’ perspectives, both pre-and post-death.

### Conclusion

Our study represents a MAID “new frontier” [30] that explores the nuances of patient experiences to inform future patients, loved ones, and clinicians as they make realistic, honest decisions about death.

## References

[1] Gawande A. Being Mortal: Medicine and What Matters in the End. Henry Holt and Company (2014). p. 304.

[2] Sallnow L Smith R Ahmedzai SH Bhadelia A Chamberlain C Cong Y Report of the Lancet Commission on the Value of Death: Bringing Death Back Into Life. Lancet (2022) 399(10327):837–84. 10.1016/S0140-6736(21)02314-X 35114146 PMC8803389

[3] Warraich H. Modern Death: How Medicine Changed the End of Life. Macmillan (2017). p. 324.

[4] Jiao K Chow AY. The Connections of Physical and Psychosocial Symptoms Among Patients With Terminal Illnesses: A Network Analysis. Palliat Med (2023) 37(1):120–30. 10.1177/02692163221128452 36474334

[5] Henson LA Maddocks M Evans C Davidson M Hicks S Higginson IJ. Palliative Care and the Management of Common Distressing Symptoms in Advanced Cancer: Pain, Breathlessness, Nausea and Vomiting, and Fatigue. J Clin Oncol (2020) 38(9):905–14. 10.1200/JCO.19.00470 32023162 PMC7082153

[6] Cassell EJ. The Nature of Suffering and the Goals of Medicine. Loss Grief Care (1998) 8(1-2):129–42. 10.1300/j132v08n01_18

[7] Malhotra C Harding R Teo I Ozdemir S Koh GCH Neo P Financial Difficulties Are Associated With Greater Total Pain and Suffering Among Patients With Advanced Cancer: Results From the COMPASS Study. Support Care Cancer (2020) 28(8):3781–9. 10.1007/s00520-019-05208-y 31832824

[8] McPherson CJ Wilson KG Murray MA. Feeling Like a Burden: Exploring the Perspectives of Patients at the End of Life. Soc Sci Med (2007) 64(2):417–27. 10.1016/j.socscimed.2006.09.013 17069943

[9] Svenaeus F. To Die Well: The Phenomenology of Suffering and End of Life Ethics. Med Health Care Philos (2020) 23(3):335–42. 10.1007/s11019-019-09914-6 31463881 PMC7426301

[10] van Hooft S. Suffering and the Goals of Medicine. Med Health Care Philos (1998) 1(2):125–31. 10.1023/a:1009923104175 11081289

[11] Kolsteren EEM Deuning-Smit E Chu AK van der Hoeven YCW Prins JB van der Graaf WTA Psychosocial Aspects of Living Long Term With Advanced Cancer and Ongoing Systemic Treatment: A Scoping Review. Cancers (2022) 14(16):3889. 10.3390/cancers14163889 36010883 PMC9405683

[12] Corpora M. The Privilege of a Good Death: An Intersectional Perspective on Dying a Good Death in America. Gerontologist (2022) 62(5):773–9. 10.1093/geront/gnab130 34467998

[13] Rowley J Richards N Carduff E Gott M. The Impact of Poverty and Deprivation at the End of Life: A Critical Review. Palliat Care Soc Pract (2021) 15:26323524211033873. 10.1177/26323524211033873 34541536 PMC8442481

[14] Stajduhar KI Mollison A Giesbrecht M McNeil R Pauly B Reimer-Kirkham S “Just Too Busy Living in the Moment and Surviving”: Barriers to Accessing Health Care for Structurally Vulnerable Populations at End-Of-Life. BMC Palliat Care (2019) 18(1):11. 10.1186/s12904-019-0396-7 30684959 PMC6348076

[15] Heyland DK Heyland R Dodek P You JJ Sinuff T Hiebert T Discordance Between Patients’ Stated Values and Treatment Preferences for End-Of-Life Care: Results of a Multicentre Survey. BMJ Support Palliat Care (2017) 7(3):292–9. 10.1136/bmjspcare-2015-001056 28827369

[16] Winter L. Patient Values and Preferences for End-Of-Life Treatments: Are Values Better Predictors Than a Living Will? J Palliat Med (2013) 16(4):362–8. 10.1089/jpm.2012.0303 23442042

[17] Gómez-Vírseda C de Maeseneer Y Gastmans C. Relational Autonomy: What Does It Mean and How Is It Used in End-of-Life Care? A Systematic Review of Argument-Based Ethics Literature. BMC Med Ethics (2019) 20(1):76. 10.1186/s12910-019-0417-3 31655573 PMC6815421

[18] Kim K Heinze K Xu J Kurtz M Park H Foradori M Theories of Health Care Decision Making at the End of Life: A Meta-Ethnography. West J Nurs Res (2018) 40(12):1861–84. 10.1177/0193945917723010 28816094 PMC6474239

[19] Levoy K Tarbi EC De Santis JP. End-of-Life Decision Making in the Context of Chronic Life-Limiting Disease: A Concept Analysis and Conceptual Model. Nurs Outlook (2020) 68(6):784–807. 10.1016/j.outlook.2020.07.008 32943221 PMC7704858

[20] Kellehear A. A Social History of Dying. Cambridge, England: Cambridge University Press (2009). 10.1017/cbo9780511481352

[21] Capron AM. Looking Back at Withdrawal of Life-Support Law and Policy to See What Lies Ahead for Medical Aid-In-Dying. Yale J Biol Med (2019) 92(4):781–91. 31866795 PMC6913806

[22] Death With Dignity. The History of the Death With Dignity Movement: 1990s to Now (2024). Available from: https://deathwithdignity.org/history/ (Accessed January 18, 2024).

[23] Oregon.gov. Oregon’s Death With Dignity Act (2024). Available from: https://www.oregon.gov/oha/PH/PROVIDERPARTNERRESOURCES/EVALUATIONRESEARCH/DEATHWITHDIGNITYACT/Pages/index.aspx (Accessed January 18, 2024).

[24] Compassion & Choices. States Where Medical Aid in Dying Is Authorized (2024). Available from: https://www.compassionandchoices.org/resource/states-or-territories-where-medical-aid-in-dying-is-authorized (Accessed January 18, 2024).

[25] Mroz S Dierickx S Deliens L Cohen J Chambaere K. Assisted Dying Around the World: A Status Quaestionis. Ann Palliat Med (2021) 10(3):3540–53. 10.21037/apm-20-637 32921084

[26] Roehr B. Assisted Dying Around the World. BMJ (2021) 374:n2200. 10.1136/bmj.n2200 34507973

[27] Kusmaul N Becker TD Gibson A Wallace CL. Medical Aid in Dying: How Might U.S. Policy Prevent Suffering at the End of Life? J Aging Soc Policy (2023) 1–18. 10.1080/08959420.2023.2226306 37348205

[28] Pope TM. Medical Aid in Dying: Key Variations Among U.S. State Laws (2021). Available from: https://papers.ssrn.com/abstract=3743855 (Accessed January 18, 2024).

[29] Kozlov E Nowels M Gusmano M Habib M Duberstein P. Aggregating 23 Years of Data on Medical Aid in Dying in the United States. J Am Geriatr Soc (2022) 70(10):3040–4. 10.1111/jgs.17925 35708060 PMC9588508

[30] Buchbinder M Cain C. Medical Aid in Dying: New Frontiers in Medicine, Law, and Culture. Annu Rev L Social Sci (2023) 19:195–214. 10.1146/annurev-lawsocsci-110722-083932

[31] Hannig A. The Day I Die: The Untold Story of Assisted Dying in America. Naperville, IL: Sourcebooks (2023).

[32] Howard M Bansback N Tan A Klein D Bernard C Barwich D Recognizing Difficult Trade-Offs: Values and Treatment Preferences for End-Of-Life Care in a Multi-Site Survey of Adult Patients in Family Practices. BMC Med Inform Decis Mak (2017) 17(1):164. 10.1186/s12911-017-0570-x 29212487 PMC5719577

[33] Supiano KP McGee N Dassel KB Utz R. A Comparison of the Influence of Anticipated Death Trajectory and Personal Values on End-Of-Life Care Preferences: A Qualitative Analysis. Clin Gerontol (2019) 42(3):247–58. 10.1080/07317115.2017.1365796 28990872

[34] Fischhoff B Barnato AE. Value Awareness: A New Goal for End-Of-Life Decision Making. MDM Policy Pract (2019) 4(1):2381468318817523. 10.1177/2381468318817523 30746498 PMC6360469

[35] Witteman HO Ndjaboue R Vaisson G Dansokho SC Arnold B Bridges JFP Clarifying Values: An Updated and Expanded Systematic Review and Meta-Analysis. Med Decis Making (2021) 41(7):801–20. 10.1177/0272989X211037946 34565196 PMC8482297

[36] Blank RH. End-of-Life Decision Making Across Cultures. J L Med Ethics (2011) 39(2):201–14. 10.1111/j.1748-720X.2011.00589.x 21561515

[37] Bullock K. The Influence of Culture on End-Of-Life Decision Making. J Soc Work End Life Palliat Care (2011) 7(1):83–98. 10.1080/15524256.2011.548048 21391079

[38] Gysels M Evans N Meñaca A Andrew E Toscani F Finetti S Culture and End of Life Care: A Scoping Exercise in Seven European Countries. PLoS One (2012) 7(4):e34188. 10.1371/journal.pone.0034188 22509278 PMC3317929

[39] Martin T Freeman S Lalani N Banner D. Qualities of the Dying Experience of Persons Who Access Medical Assistance in Dying: A Scoping Review. Death Stud (2023) 47(9):1033–43. 10.1080/07481187.2022.2160033 36579696

[40] Nuhn A Holmes S Kelly M Just A Shaw J Wiebe E. Experiences and Perspectives of People Who Pursued Medical Assistance in Dying: Qualitative Study in Vancouver, BC. Can Fam Physician (2018) 64(9):e380–6. 30209111 PMC6135118

[41] Bullock EC. Assisted Dying and the Proper ROLE of Patient Autonomy. In: Cholbi M Varelius J, editors. New Directions in the Ethics of Assisted Suicide and Euthanasia. Cham: Springer International Publishing (2023). p. 1–16.

[42] Colburn B. Autonomy, Voluntariness and Assisted Dying. J Med Ethics (2020) 46(5):316–9. 10.1136/medethics-2019-105720 31719156

[43] Pesut B Wright DK Thorne S Hall MI Puurveen G Storch J What’s Suffering Got to Do with it? A Qualitative Study of Suffering in the Context of Medical Assistance in Dying (MAID). BMC Palliat Care (2021) 20(1):174. 10.1186/s12904-021-00869-1 34758799 PMC8582137

[44] Güth U Weitkunat R McMillan S Schneeberger AR Battegay E. WHEN the Cause of Death Does Not Exist: Time for the WHO to Close the ICD Classification Gap for Medical Aid in Dying. EClinicalMedicine (2023) 65:102301. 10.1016/j.eclinm.2023.102301 38021370 PMC10660015

[45] Oczkowski SJW Crawshaw DE Austin P Versluis D Kalles-Chan G Kekewich M How Can We Improve the Experiences of Patients and Families Who Request Medical Assistance in Dying? A Multi-Centre Qualitative Study. BMC Palliat Care (2021) 20(1):185. 10.1186/s12904-021-00882-4 34876104 PMC8653618

[46] Merleau-Ponty M. Phenomenology of Perception. New York, NY: Humanities Press (1962).

[47] Carel H. Phenomenology of Illness. London, England: Oxford University Press (2016).

[48] Dahlberg K. The Essence of Essences – The Search for Meaning Structures in Phenomenological Analysis of Lifeworld Phenomena. Int J Qual Stud Health Well-being (2006) 1(1):11–9. 10.1080/17482620500478405

[49] Finlay L. Unfolding the Phenomenological Research Process: Iterative Stages of “Seeing Afresh”. J Humanistic Psychol (2013) 53(2):172–201. 10.1177/0022167812453877

[50] Wertz FJ. Phenomenological Research Methods for Counseling Psychology. J Couns Psychol (2005) 52(2):167–77. 10.1037/0022-0167.52.2.167

[51] Wertz FJ. Five Ways of Doing Qualitative Analysis: Phenomenological Psychology, Grounded Theory, Discourse Analysis, Narrative Research, and Intuitive Inquiry. New York, NY: Guilford Press (2011). p. 434.

[52] Ashworth PD. Seeing Oneself as a Carer in the Activity of Caring: Attending to the Lifeworld of a Person With Alzheimer’s Disease. Int J Qual Stud Health Well-being (2006) 1(4):212–25. 10.3402/qhw.v1i4.4935

[53] Padgett D. Qualitative Methods in Social Work Research. Thousand Oaks, CA: SAGE (2008). p. 281.

[54] Tong A Sainsbury P Craig J. Consolidated Criteria for Reporting Qualitative Research (COREQ): A 32-Item Checklist for Interviews and Focus Groups. Int J Qual Health Care (2007) 19(6):349–57. 10.1093/intqhc/mzm042 17872937

[55] Software VE. MAXQDA 2022 (2024). Available from: https://www.maxqda.com/help-mx22/welcome (Accessed January 18, 2024).

[56] Hennink MM Kaiser BN Marconi VC. Code Saturation Versus Meaning Saturation: How Many Interviews Are Enough? Qual Health Res (2017) 27(4):591–608. 10.1177/1049732316665344 27670770 PMC9359070

[57] Saunders B Sim J Kingstone T Baker S Waterfield J Bartlam B Saturation in Qualitative Research: Exploring its Conceptualization and Operationalization. Qual & Quantity (2017) 52(4):1893–907. 10.1007/s11135-017-0574-8 PMC5993836 29937585

[58] Hossain MS Alam MK Ali MS. Phenomenological Approach in the Qualitative Study: Data Collection and Saturation. ICRRD Qual Index Res J (2024) 5(2). 10.53272/icrrd.v5i2.4

[59] Buchbinder M. Choreographing Death: A Social Phenomenology of Medical Aid-In-Dying in the United States. Med Anthropol Q (2018) 32(4):481–97. 10.1111/maq.12468 30014621

[60] Cain CL McCleskey S. Expanded Definitions of the “Good Death”? Race, Ethnicity and Medical Aid in Dying. Sociol Health Illn (2019) 41(6):1175–91. 10.1111/1467-9566.12903 30950077 PMC6786270

[61] Buchbinder M. Dirty Work in Medicine: Understanding U.S. Physicians’ Agency in Contested Medical Practices. Med Anthropol Q (2022) 36(4):534–51. 10.1111/maq.12720 35986924

[62] Scambler G. Health-Related Stigma. Sociol Health Illn (2009) 31(3):441–55. 10.1111/j.1467-9566.2009.01161.x 19366430

[63] Ellingsen S Roxberg Å Kristoffersen K Rosland JH Alvsvåg H. Entering a World With No Future: A Phenomenological Study Describing the Embodied Experience of Time When Living With Severe Incurable Disease. Scand J Caring Sci (2013) 27(1):165–74. 10.1111/j.1471-6712.2012.01019.x 22708714

[64] Moskalewicz M Popova Y Wiertlewska-Bielarz J. Lived Time in Ovarian Cancer - A Qualitative Phenomenological Exploration. Eur J Oncol Nurs (2022) 56:102083. 10.1016/j.ejon.2021.102083 34998214

[65] Rovers JJE Knol EJ Pieksma J Nienhuis W Wichmann AB Engels Y. Living at the End-Of-Life: Experience of Time of Patients With Cancer. BMC Palliat Care (2019) 18(1):40. 10.1186/s12904-019-0424-7 31088442 PMC6518794

[66] Pottash M Saikaly K Stevenson M Krohmal B. A Survey of Clinicians Who Provide Aid in Dying. Am J Hosp Palliat Care (2023):10499091231205841. 10.1177/1049909123120584 37776055

[67] Davidson JE Stokes L DeWolf Bosek MS Turner M Bojorquez G Lee YS Nurses’ Values on Medical Aid in Dying: A Qualitative Analysis. Nurs Ethics (2022) 29(3):636–50. 10.1177/09697330211051029 35104169

[68] Zhou YMJ Shelton W. Physicians’ End of Life Discussions With Patients: Is There an Ethical Obligation to Discuss Aid in Dying? HEC Forum (2020) 32(3):227–38. 10.1007/s10730-020-09402-y 32221816

[69] Thangarasa T Hales S Tong E An E Selby D Isenberg-Grzeda E A Race to the End: Family Caregivers’ Experience of Medical Assistance in Dying (MAiD)-A Qualitative Study. J Gen Intern Med (2022) 37(4):809–15. 10.1007/s11606-021-07012-z 34287775 PMC8904693

[70] Lowers J Scardaville M Hughes S Preston NJ. Comparison of the Experience of Caregiving at End of Life or in Hastened Death: A Narrative Synthesis Review. BMC Palliat Care (2020) 19(1):154. 10.1186/s12904-020-00660-8 33032574 PMC7545566

[71] Frolic AN Swinton M Murray L Oliphant A. Double-Edged MAiD Death Family Legacy: A Qualitative Descriptive Study. BMJ Support Palliat Care (2020) 18:e845–50. 10.1136/bmjspcare-2020-002648 33355177

[72] Gamondi C Fusi-Schmidhauser T Oriani A Payne S Preston N. Family Members’ Experiences of Assisted Dying: A Systematic Literature Review With Thematic Synthesis. Palliat Med (2019) 33(8):1091–105. 10.1177/0269216319857630 31244384

[73] Buchbinder M. Scripting Death: Stories of Assisted Dying in America. Oakland, CA: Univ of California Press (2021). p. 248.

[74] Krinsky J. Embracing the End: A Comparative Analysis of Medical Aid in Dying in Canada and the United States. Brook J Int’l L (2022) 48:331.

[75] Quah ELY Chua KZY Lua JK Wan DWJ Chong CS Lim YX A Systematic Review of Stakeholder Perspectives of Dignity and Assisted Dying. J Pain Symptom Manage (2023) 65(2):e123–36. 10.1016/j.jpainsymman.2022.10.004 36244639

[76] Variath C Peter E Cranley L Godkin D Just D. Relational Influences on Experiences With Assisted Dying: A Scoping Review. Nurs Ethics (2020) 27(7):1501–16. 10.1177/0969733020921493 32436431

[77] Mann G. A Good Death: End-Of-Life Lawyering Through a Relational Autonomy Lens. Wash L Rev. (2023) 98(4):1259.

[78] Byrnes E Ross AI Murphy M. A Systematic Review of Barriers and Facilitators to Implementing Assisted Dying: A Qualitative Evidence Synthesis of Professionals’ Perspectives. Omega (2022) 003022282211166. 10.1177/00302228221116697 35929771

[79] Buchbinder M. Access to Aid-In-Dying in the United States: Shifting the Debate From Rights to Justice. Am J Public Health (2018) 108(6):754–9. 10.2105/AJPH.2018.304352 29672149 PMC5944872

[80] Pope T. Top Ten New and Needed Expansions of U.S. Medical Aid in Dying Laws. Amer J Bioeth (2023) 23(11):89–91. 10.1080/15265161.2023.2256244 37879023

